# Vitamin D_3_ promotes white fat beige through IL-27/P38MAPK/PGC-1α pathway

**DOI:** 10.3389/fnut.2025.1661072

**Published:** 2025-09-18

**Authors:** Yanqiu Zhou, Junfang Shu, Yueying Zhao, Xiaorong Wu, Zhijun He, Xinzhe Lyu, Yong Zhou, Ling Ma

**Affiliations:** ^1^Department of Nutrition and Food Hygiene, School of Public Health, Southwest Medical University, Luzhou, China; ^2^Department of Clinical Nutrition, Chongqing General Hospital, Chongqing University, Chongqing, China; ^3^School of Public Health, Southwest Medical University, Luzhou, China; ^4^Department of Medical Cell Biology and Genetics, School of Basic Medical Science, Southwest Medical University, Luzhou, China; ^5^Environmental Health Effects and Risk Assessment Key Laboratory of Luzhou, School of Public Health, Southwest Medical University, Luzhou, China

**Keywords:** vitamin D_3_, white fat beige, obesity, IL-27, PGC-1α, UCP-1

## Abstract

**Background:**

Obesity is turning into a more critical problem for public health. Vitamin D_3_ (VD_3_) may be strongly linked to obesity.

**Objectives:**

The study aims to examine the influence of VD_3_ on IL-27 levels and the molecular mechanism by which VD_3_ affects white fat beige through the IL-27/P38MAPK/PGC-1α pathway.

**Methods:**

Firstly, a small sample population study was conducted to compare the disparities in serum 25(OH)D_3_ and IL-27 between individuals with obesity and healthy control groups. Secondly, twenty-four Wistar rats were separated into three groups: CON, HFD, and HFD + VD_3_ groups. Following 7 weeks of intervention, detection of biochemical indicators in serum by enzyme-linked immunosorbent assay (ELISA), mRNA, and protein expression of vitamin D receptor (VDR), IL-27R, P38MAPK, PGC-1α, and UCP-1 in inguinal adipose tissue by RT-qPCR and western blot. Finally, 3T3-L1 cells were induced into a hypertrophic adipose model, knock down IL-27 or PGC-1α using small interfering RNA, treated with 100 nM Calcitriol for 24 h, and divided into CON, PA, PA + 1,25(OH)_2_D_3_, PA + si IL-27, PA + si IL-27 + 1,25(OH)_2_D_3,_ PA + si PGC-1α, and PA + si PGC-1α + 1,25(OH)_2_D_3_ groups. Detection of TC, TG, and IL-27 levels by ELISA, mRNA, and protein expression of VDR, IL-27R, P38MAPK, PGC-1α, UCP-1, and CD137 in cell supernatant by RT-qPCR and western blot.

**Results:**

A correlation was identified between serum 25(OH)D_3_ and IL-27 in the population-based study. However, no statistically significant difference in serum 25(OH)D_3_ or IL-27 levels was observed between the observation group and the control group. After VD_3_ intervention, TC, TG, and the number of LDs were significantly reduced in both HFD rats and 3T3-L1 cells, and serum IL-6 and MCP-1 in HFD rats were decreased. Meanwhile, there was a significant increase in mRNA and protein expression for VDR, IL-27R, P38MAPK, and PGC-1α. The expressions of the UCP-1 protein and the CD137 mRNA dramatically increased. Knockdown of IL-27 eliminated the increasing effect of calcitriol on the expression of P38MAPK, PGC-1α, UCP-1, and CD137 in 3T3-L1 cells, and knockdown of PGC-1α eliminated the increasing effect of calcitriol on the expression of UCP-1 and CD137 in 3T3-L1 cells.

**Conclusion:**

The study shows that VD_3_ may promote white fat beige through the IL-27/P38MAPK/PGC-1α pathway.

## 1 Introduction

Obesity is characterized by an excessive accumulation of body fat, particularly in specific regions. The imbalance between energy intake and expenditure in adipose tissue leads to weight gain, ultimately resulting in obesity. In recent decades, mammalian adipose tissue has been broadly classified into three categories: white adipose tissue (WAT), brown adipose tissue (BAT), and beige adipose tissue (1). White adipocytes function primarily as energy reservoirs, whereas brown adipocytes act as energy consumers. Certain progenitor cells and white adipocytes located in WAT can undergo a process known as “browning” when exposed to specific stimuli such as cold exposure, exercise, and pharmacological interventions (2, 3). Active beige adipose tissue is capable of generating substantial heat through the production of a key protein called mitochondrial uncoupling protein 1 (UCP-1) (4). Elevated levels of free fatty acids can enhance UCP-1 protein expression, prompting beige adipose tissue to metabolize a significant amount of fatty acids, thereby facilitating their clearance. Consequently, the activation of beige fat is anticipated to emerge as a novel therapeutic target for weight loss and the amelioration of related metabolic disorders and inflammatory responses (5).

The effect of nutrients on gene expression is termed nutrigenomics. This field concentrates on understanding how dietary components influence gene expression through epigenetic mechanisms. Nutrients found in the diet, such as polyphenols, fatty acids, and vitamins, can modulate epigenetic markers, including DNA methylation, histone modifications, and non-coding RNAs. These modifications subsequently impact gene expression related to glucose metabolism, lipid homeostasis, and inflammation. Nutrigenomics offers a scientific foundation for personalized nutritional interventions by examining the interactions between genes and nutrients, thereby playing a crucial role in managing chronic diseases, such as obesity and related metabolic syndromes (6).

Interleukin-27 (IL-27), a member of the IL-12 family, is an exogenous dimeric cytokine (7). IL-27 has a direct inhibitory effect on *γ* delta T17 cells, a class of innate immune cells that respond rapidly to inflammation. By activating the STAT1 pathway, IL-27 inhibits IL-17 production in γ delta T17 cells, thereby alleviating inflammation (8). A research team discovered that the overexpression of IL-27 promotes the expression of key thermogenic genes in brown adipose tissue, while simultaneously reducing chronic inflammation and macrophage infiltration within white adipose tissue (9). IL-27 and IL-27R interact to initiate the activation of the p38 MAPK signaling pathways (10). The p38MAPK pathway serves a crucial function in the differentiation of fat precursor cells. TAK-715 (p38 inhibitor) significantly inhibits adipocyte differentiation, reduces intracellular TG and lipid accumulation, and the p38MAPK pathway directly regulates adipogenesis (11). After IL-27 intervention, the key transcription activators PGC-1α and UCP-1, which control energy metabolism in beige adipocytes, were significantly upregulated (9). CD137 is one of the key biomarkers for the differentiation of beige adipocytes, and its expression changes are closely related to the process of beige (12, 13). UCP-1, PGC-1α, and CD137 expressions suggest the existence of beige adipose-like cells within white adipose tissue, which enable adaptive heat production and facilitate energy expenditure.

Furthermore, emerging evidence suggests that micronutrients may exert an effect on body weight regulation, adipocyte differentiation, and the transformation of white adipocytes into brown or beige adipocytes (14). Vitamin D_3_ (VD_3_) is a lipophilic hormone derived from cholesterol, with its main active form being 1,25(OH)_2_D_3_ (15). VD_3_ is not only involved in the regulation of calcium and phosphorus balance but is also closely related to inflammation, immune regulation, energy metabolism, and fat production (16). Vitamin D can directly inhibit inflammation in adipose tissue and reduce the expression of pro-inflammatory factors such as TNF-*α* and IL-6 (17). Vitamin D may partially mitigate the cancer-promoting effects of obesity by modulating mechanisms such as inflammation, insulin resistance, and oxidative stress (18). A large population study from China found that children and adolescents with vitamin D deficiency were significantly more susceptible to obesity and metabolically unhealthy obesity (19). A comprehensive meta-analysis demonstrated that individuals with obesity have a 35% greater likelihood of experiencing vitamin D_3_ deficiency when compared to individuals with normal weight, and a 24% higher risk when compared to those who are overweight (20). A wealth of evidence suggests that VD_3_ represents a viable target for food-based nutritional interventions (21–24). Chen et al. found that vitamin D_3_ supplementation has the potential to modulate mitochondrial function and improve tubulointerstitial fibrosis in diabetic rats (25). Results from Xiang’s team suggest that VD_3_ supplementation can alter the composition of the gut microbiota in mice and improve parameters of obesity caused by HFD (26).

Previous studies have reported that 1,25(OH)_2_D_3_ can impede lipid droplet (LD) fusion, promote LD decomposition, reduce LD volume, and inhibit lipogenesis in 3T3-L1 cells via the PPAR-*α* signaling pathway (27). However, the effects of vitamin D_3_ on white fat beige and the specific molecular mechanisms involved remain to be further explored. Therefore, this study aims to investigate the correlation between 25(OH)D_3_ and IL-27 in a small-sample population. Additionally, by constructing high-fat diet Wistar rat and 3T3-L1 mature adipocyte models, we explored the effects of VD_3_ on IL-27 levels and the specific mechanisms by which VD_3_ regulates energy metabolism and promotes white fat beige through the IL-27/P38MAPK/PGC-1α pathway.

## 2 Materials and methods

### 2.1 Population trial design

In the Introduction of the Recommended Opinions on Chinese Adult Body Mass Index Classification (28), the proposed body mass index (BMI) standards for Chinese individuals are as follows: “healthy weight” is defined as a BMI of 18.5 kg/m^2^ ≤ BMI < 24 kg/m^2^; “Overweight” is classified as a BMI of 24 kg/m^2^ ≤ BMI < 28 kg/m^2^; and “Obese” is defined as a BMI of 28 kg/m^2^ or greater. For the observation group (people with overweight and obesity), the inclusion criteria were as follows: ① age ranging from 18 to 45 years and ② BMI ≥ 24 kg/m^2^. The control group inclusion criteria were: ① age ranging from 18 to 45 years and ② BMI ranging from 18.5 kg/m^2^ to < 24 kg/m^2^. Participants were excluded if they met any of the following conditions: individuals with diabetes; patients who had taken diet pills within the past 3 months; individuals who had received vitamin D supplementation or complex preparations within the past 3 months; individuals with osteoporosis and autoimmune diseases. Previous studies have indicated that the difference of $\bar{x}$ *±s* of serum 25(OH)D_3_ level between healthy people and people with overweight and obesity was 6.4 ± 8.71. The minimum sample size required for each group was 31 cases by means of comparison of two independent samples using PASS 15.0 software. Eventually, the trial included 64 individuals, where 33 were designated for the observation group and 31 for the control group.

Participants were recruited from a local university through posters and online platforms. Following an overnight fast of 8 hours, physiological parameters were assessed, and approximately 3–5 mL of blood was drawn from the median cubital vein of the volunteers for serological analysis. This case–control study received ethical approval from the Research Ethics Committee of Southwest Medical University, and written informed consent was obtained from each participant (Reference Number: SWMUIRBKS-202307-0007).

### 2.2 Human serological test

Following an overnight fast of at least 8 hours, all participants underwent a comprehensive medical evaluation in the morning. A trained nurse measured and documented their age, height, weight, BMI, and waist circumference. Fasting venous blood samples were collected, and serum was separated through centrifugation. Enzyme-linked immunosorbent assay (ELISA) was utilized to analyze the serum levels of 25(OH)D_3_, IL-27, TG, TC, HDL-C, LDL-C, and GLU. The kits were sourced from Mlbio (Mlbio, China) and Jiancheng (Jiancheng Bio, China).

### 2.3 Animals

Five-week-old male Wistar rats (140-150 g) were bought from Dossy Experimental Animal Co., Ltd. (Chengdu, Sichuan, China). The rats were maintained in a controlled environment at a temperature of 23 ± 1°C and a humidity of 50 ± 10%, with a 12-h light/dark cycle, permitting ad libitum access to food and water. A total of 24 rats were randomly assigned to two groups: the CON group (*n* = 8), which received standard feed for 14 weeks, and the high-fat feed group (*n* = 16), which was administered high-fat feed for 14 weeks. At week 7, 16 rats in the HFD group were weighing 20% higher than the mean weight in the NCD group and randomly partitioned into the HFD group (*n* = 8) and the HFD + VD group (*n* = 8) for a 7-week intervention. In the HFD + VD group, cholecalciferol (1,25(OH)D_3_; Sigma, Germany) was solubilized in maize oil (Aladdin, China) at a concentration of 2.5 mg/mL, administered intragastrically at a dosage of 12.5 mg/kg (29, 30). During the experiment, the rats’ food intake was recorded every day, and their weight was measured and recorded every week. At the end of the 14th week, the rats were anesthetized with pentobarbital sodium (45 mg·kg^−1^·BW) and killed. Blood was collected from the heart and centrifuged at 4000 rpm for 15 min. Then, serum samples were extracted for further examination. Inguinal adipose tissue was removed from each animal, weighed immediately, and stored at −80°C. The Lee’s index was calculated as follows: [(weight)⅓/body length ×1,000]. All sampling processes were carried out in an SPF environment. All animal research procedures strictly adhere to the 1996 National Institutes of Health Guidelines for the Care and Use of Laboratory Animals. The experimental protocols conducted in this study have received approval from the Ethics Committee of Southwest Medical University, in accordance with established guidelines (Approval No. 2020612; Date of approval: August 17, 2020).

### 2.4 Cell culture

3T3-L1 adipocytes were cultured in DMEM supplemented with 10% fetal bovine serum (FBS; Cellmax, Australia). The cells were maintained in a standard cell culture incubator at 37°C with 5% CO_2_. To induce differentiation in the 3T3-L1 cells, the “cocktail method” was employed. The cells were seeded into 6-well plates and allowed to reach confluency. Two days after reaching confluency, the growth media was supplemented with 10 μg/mL insulin (Sigma-Aldrich, Germany), 1 μmol/L dexamethasone (Sigma-Aldric, Germany), and 0.5 mmol/L 1-methyl-3-isobutyl-xanthine (Sigma-Aldrich, Germany). The differentiation process continued for 2 days. From Days 4 to 7, the cells were maintained in growth media supplemented with 10 μg/mL insulin. Subsequently, the mature adipocytes were cultured in regular growth media. Following 8–10 days of culture, the cells reached a mature adipocyte state. Building upon this foundation, a model of hypertrophic adipocytes was established by intervening with 300 μM palmitic acid (PA; Pythonbio, China) for 24 h. After this intervention, the research group further intervened with 100 nM calcitriol (MCE, USA) for an additional 24 h (27).

### 2.5 Small-interfering RNA (siRNA) transfection

RNA oligonucleotides targeting IL-27 (palindromic sequence: CAAUCAGGUGUCAUCCCAA) and PGC-1α (palindromic sequence: GUAGCGACCAAUCGGAAAUTT) were procured from TSINGKE (Beijing, China) and GenePharma (Shanghai, China) to facilitate the silencing of the target proteins. Negative control siRNAs, which exhibited no homology with the mouse genome, were also obtained from TSINGKE and GenePharma. The 3T3-L1 cells were transfected with siRNA utilizing Lipofectamine 2000 (Invitrogen, USA). Following the establishment of the cells as a model for hypertrophic adipocytes, the aforementioned siRNA (2.5 μg) and 5 μL of Lipofectamine 2000 reagent were diluted in 250 μL of Opti-MEM medium (BasalMedia, China) and incubated separately for 5 min at room temperature. After this incubation period, equal volumes of the diluted siRNA and Lipofectamine 2000 reagent were gently mixed and allowed to incubate for an additional 15 min at room temperature to form siRNA-lipid complexes. For transfection, these siRNA-lipid complexes were subsequently incubated with the 3T3-L1 cells in 6-well culture plates for 24 h. Following transfection, the cells were treated with 100 nM calcitriol (MCE, USA) for 24 h and subsequently harvested.

### 2.6 ORO staining of frozen section and 3T3-L1 cells

A cryotome was used to cut 22 μm sections of frozen adipose tissue, which were then immersed in phosphate buffer solution (PBS) for 10 s, followed by three consecutive rinses in distilled water, each lasting 2 s. The samples were subsequently soaked in 60% isopropyl alcohol for 5 min and rinsed. They were then dipped in oil red O staining solution for 10 min, followed by a brief wash in distilled water for 2 s, and finally encased in glycerin gelatin. Images were captured using a microscope (Motic, China).

After being washed twice with phosphate-buffered saline (PBS; Solarbio, China), 3T3-L1 cells were fixed in 4% paraformaldehyde for 30 min. Following fixation, the cells were washed with a detergent for 30 s and then incubated with a refined Oil Red O (ORO) working solution (Beyotime, China).

### 2.7 Biochemical analysis

The serum levels of TG, TC, HDL-C, LDL-C, and GLU were measured using an automatic biochemical analyzer (Olympus, Japan).

### 2.8 ELISA analysis

The serum levels of TNF-α (NeoBioscience, China), MCP-1 (NeoBioscience, China), IL-6 (NeoBioscience, China), IL-27 (Mlbio, China), and 25(OH)D_3_ (Elabscience, China) were quantified using ELISA kits according to the manufacturer’s instructions. Homogenize the inguinal adipose tissue in working fluid, 12,000 × g, Centrifuge for 10 min, remove the supernatant, and repeat this process twice. The TG content and TC content of adipose tissue were tested according to the production manual. TC and TG in the cell lysate were detected. After VD_3_ intervention, the cell supernatant was centrifuged, and the levels of IL-27 (Mlbio, China) were detected through ELISA.

### 2.9 Western blot analysis

Total protein was extracted from inguinal adipose tissue and 3T3-L1 cells, and the concentration was measured using the bicinchoninic acid (BCA) method (Solarbio, China). Protein was separated by 10% sodium dodecyl sulfate polyacrylamide gel electrophoresis (Solarbio, China) and transferred to polyvinylidene fluoride (PVDF) membrane (Millipore, USA). The membranes were blocked with 5% skim milk (Solarbio, China) containing 0.1% Tween® 20 (Solarbio, China) for 2 h. After washing with 0.1% tris buffer saline 20 (TBST), incubate the membrane with specific antibodies overnight. On the second day after TBST washing, incubate with antibody (CST, USA) conjugated with rabbit immunoglobulin G (IgG) and horseradish peroxidase (HRP) at a dilution of 1:2000 for 1 h. Protein expression was detected using the GelView system (BLT, China), and immune response analysis was performed using ImageJ (ImageJ 1.45 s, USA). The specific antibodies were against vitamin D receptor (VDR) (Abcam, UK), IL-27R (ABclonal, Chinese), P38MAPK (CST, USA), PGC-1α (Affinity, China), and UCP-1 (Abcam, UK). *β*-actin (CST, USA) and GAPDH (CST, USA) were used as internal references.

### 2.10 RT-qPCR

Total RNA was isolated from frozen inguinal adipose tissue (rats) or 3T3-L1 cells using RNAiso Plus (Takara, Japan). The purity and concentration of total RNA were measured using an Ultra-Micro Nucleic Acid Assay (ThermoFisher, USA) and PrimeScript RT kit (Takara, Japan) to reverse transcribe the total RNA into cDNA. Purified TB green fluorescent dye mixture (Takara, Japan) was used to conduct RT qPCR analysis in a real-time fluorescence quantitative PCR instrument (qTOWER3G, Analytical Jena) according to the protocol. The relative expression levels of the genes were calculated with the 2^−ΔΔCt^ method, using *β*-actin as an endogenous reference. The sequences of the primers were listed in Supplementary Table 1.

### 2.11 Statistical analysis

Statistical analyses were performed with SPSS 26.0 (SPSS Inc., USA) and GraphPad 10 (GraphPad Software, USA). Data are shown as the mean ±SEM. Univariate analysis of variance (ANOVA) was used to evaluate the differences among the three groups. When *p* < 0.05, the Dunnett multiple comparison method was used to compare the differences between the two groups. The correlation between human serum 25(OH)D_3_ and IL-27 was evaluated using the Spearman correlation method. Statistical significance was set at *p* < 0.05 (two-tailed).

## 3 Results

### 3.1 The fundamental information and biochemical parameters of the participants

**Figure 1:**
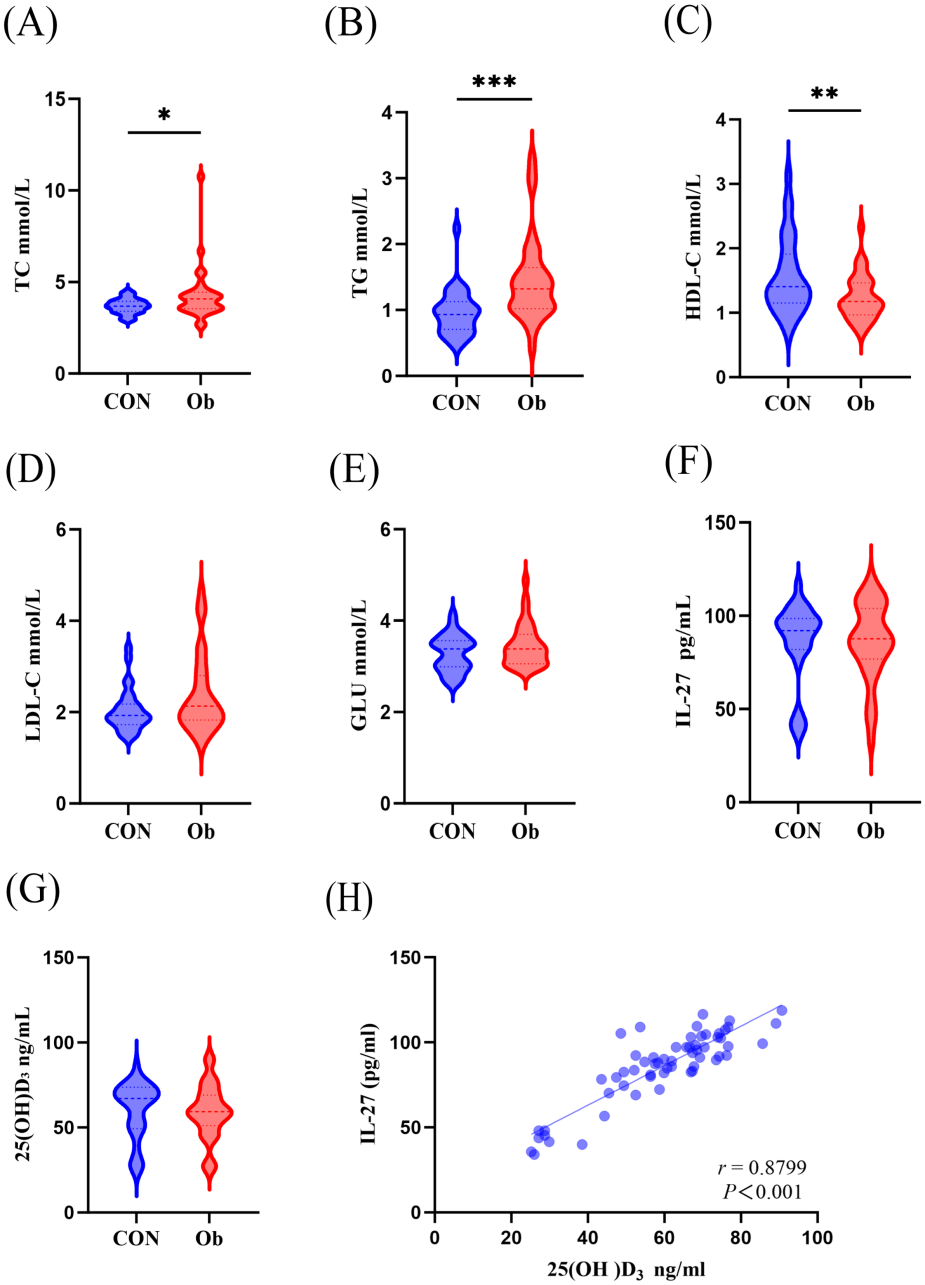
Comparison of serum biochemical indicators between the observation group (people with overwight/obese) and the control group. **(A)** Serum TC, Total cholesterol, **(B)** TG, Triglyceride, **(C)** HDL-C, High density lipoprotein cholesterol, **(D)** LDL-C, Low density lipoprotein cholesterol, **(E)** GLU, Glucose, **(F)** IL-27, Interleukin-27, **(G)** 25(OH)D_3_ levels. **(H)** Correlation analysis of serum 25(OH)D_3_ and IL-27. Data were shown as the mean ±SEM. (*n* = 64) *p < 0.05, **p < 0.01, ***p < 0.001.

Listed in Table 1 are the fundamental information and biochemical parameters of the participants. Compared with the control group, the BMI, WC, TC, and TG of the participants in the observation group were significantly increased, and HDL-C was significantly decreased (Table 1; Figures 1A–C). The two groups showed no disparity in serum LDL-C and GLU (Table 1; Figures 1D–E).

**Table 1:** The fundamental information and biochemical parameters of observation and controls.

Variable	Observation *n*=33	Control *n*=31	*t / χ^2^*	*p*
Age (year)	24.30±4.23	23.68±2.06	*−*0.745	0.459
Sex (male)	19 (57.60%)	17 (54.80%)	0.049	0.825
BMI (kg/m^2^)	27.09±2.42	20.77±1.39	*−*12.680	<0.05
WC (cm)	86.89±9.36	73.36±6.08	*−*6.811	<0.05
TC (mmol/L)	4.27±1.45	3.69±0.41	2.149	0.036
LDL-C (mmol/L)	2.38±0.86	2.04±0.47	1.838	0.072
GLU (mmol/L)	3.46±0.47	3.28±0.39	1.634	0.107
IL-27 (pg/ML)	86.23±20.92	85.65±21.91	1.104	0.917
25(OH)D_3_ (ng/ML)	59.10±16.04	60.42±16.57	*−*0.315	0.754

### 3.2 IL-27 is positively correlated with 25(OH)D_3_

There were no changes in serum 25(OH)D_3_ and IL-27 levels between the two groups (Figures 1F,G), but interestingly, a positive correlation was observed between serum 25(OH)D_3_ and IL-27 levels (Figure 1H).

### 3.3 VD_3_ reduces fat accumulation and inflammatory secretion *in vivo*

**Figure 2:**
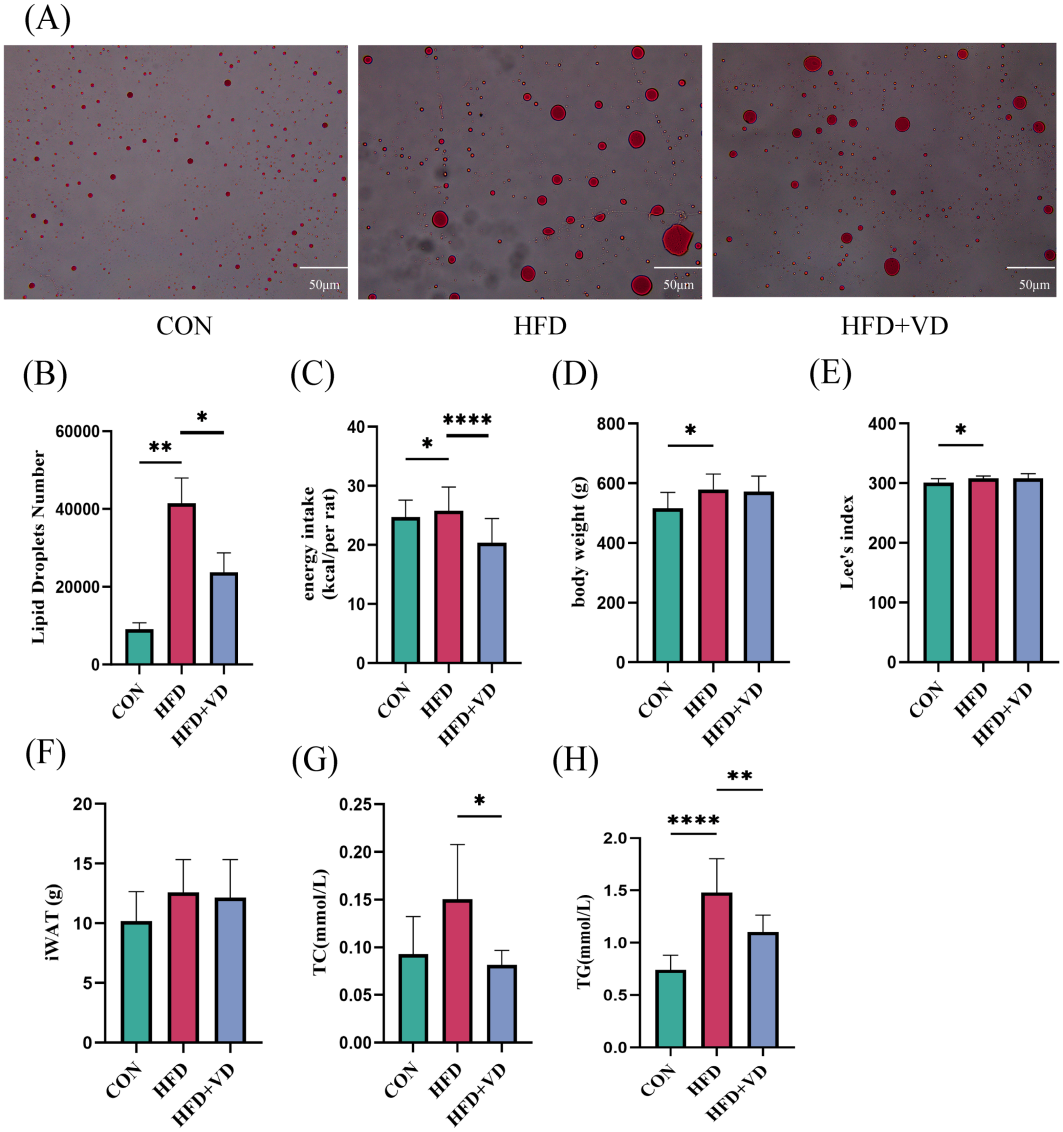
Vitamin D_3_ intervention improved lipid deposition in rats on a high fat diet. **(A)** Oil red O staining of white adipose tissue in the groin (400 × magnification). **(B)** Number of LDs. **(C)** Energy intake. **(D)** Body weight. **(E)** Lee’s index, **(F)** Inguinal adipose tissue. **(G)** TC, **(H)** TG levels in inguinal adipose tissue. Data were shown as the mean ±SEM. *p < 0.05, **p < 0.01, ****p < 0.0001.

**Figure 3:**
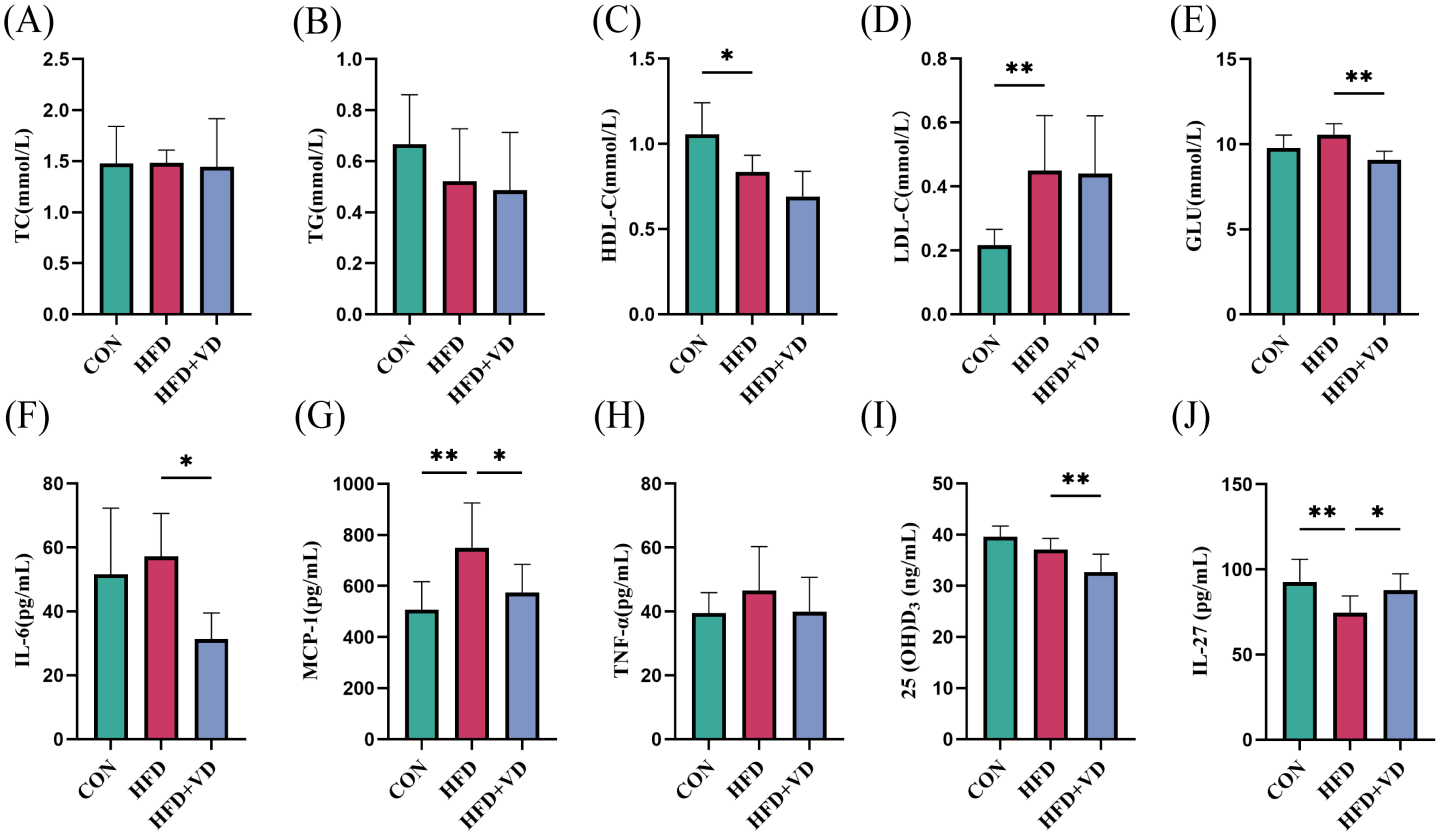
Effects of Vitamin D_3_ on levels of serum lipids, inflammatory factors, 25(OH)D_3_ and IL-27 in high-fat feeding rats. **(A)** Serum TC, **(B)** TG, **(C)** HDL-C, **(D)** LDL-C, **(E)** GLU, **(F)** IL-6, **(G)** MCP-1, **(H)** TNF-α, **(I)** 25(OH)D_3_,**(J)** IL-27 levels. Data were shown as the mean ±SEM. *p < 0.05, **p < 0.01, ***p < 0.001, ****p < 0.0001.

The results showed that compared to the CON rats, the levels of large round transparent LD, TC, and TG in the inguinal adipose tissue of rats induced by HFD were significantly increased. Following intervention with VD_3_, the size of LDs in the adipose tissue was reduced, and there was a notable decrease in TC and TG levels (Figures 2A,B,G,H). Additionally, HFD rats exhibited significantly increased energy intake and Lee’s index compared to CON rats; however, after VD_3_ treatment, energy intake was substantially reduced. Notably, there were no significant changes observed in body weight, Lee’s index, or iWAT following VD_3_ supplementation (Figures 2C–F). Furthermore, serum TC, TG, GLU, IL-6, and TNF-*α* of rats in the HFD group were not obviously changed, while LDL-C and MCP-1 were sharply improved. Conversely, the serum HDL-C, 25(OH)D_3_, and IL-27 were remarkably reduced (Figures 3A–J). Following VD_3_ intervention, serum GLU, IL-6, MCP-1, and 25(OH)D_3_ were significantly decreased (Figures 3A–I), while the serum IL-27 level of HFD rats was significantly increased (Figure 3J).

### 3.4 VD_3_ promotes white fat beige in HFD rats

**Figure 4:**
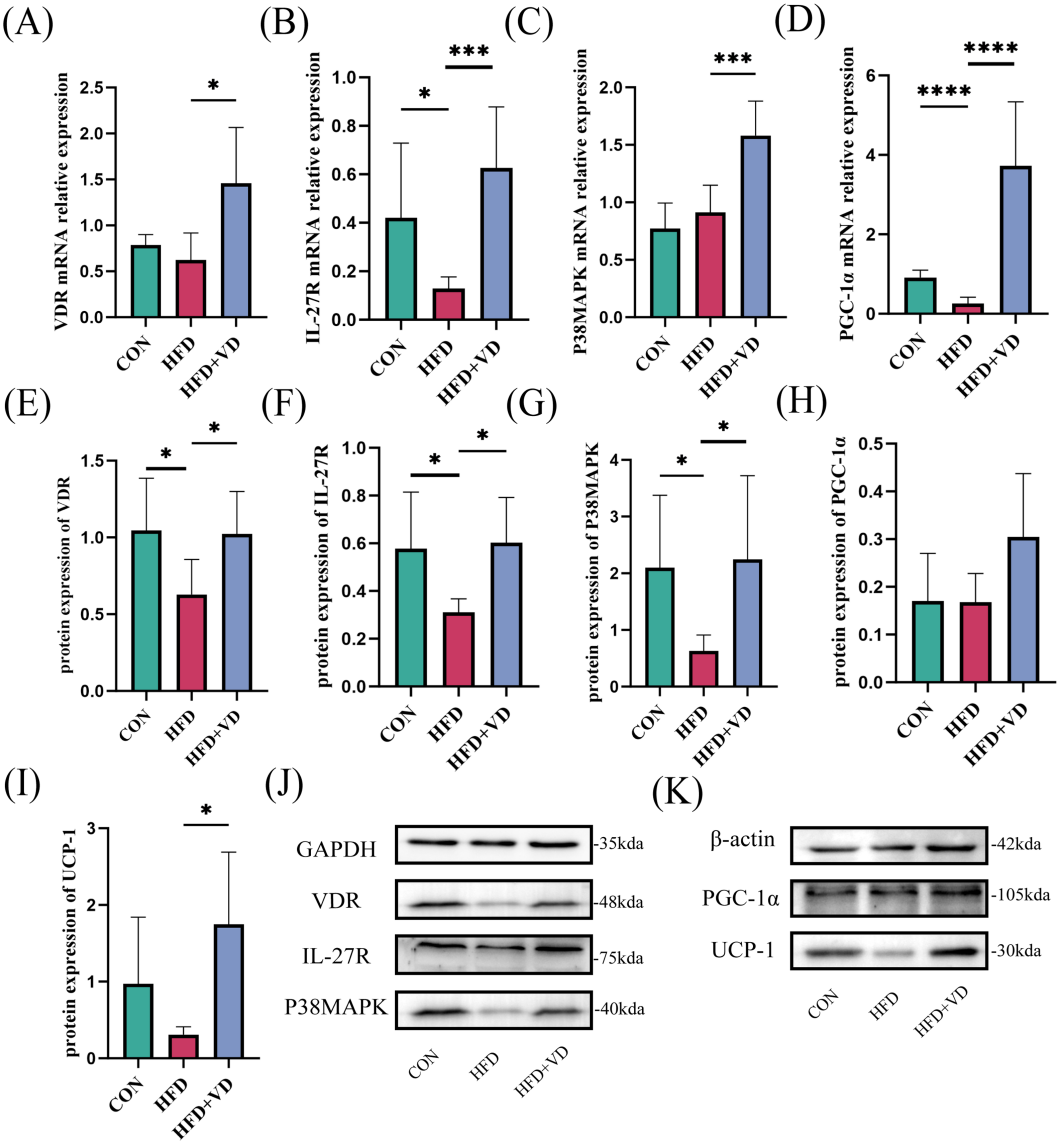
Effects of vitamin D_3_ on mRNA and protein levels of IL-27/P38MAPK/PGC-1α pathway in high-fat diet rats. **(A–D)** The mRNA expression of VDR, IL - 27R, P38MAPK, and PGC-1α were detected by qPCR, **(E–K)** The protein expression of VDR, IL-27R, P38MAPK, PGC-1α, and UCP-1 were detected by western blot, Data were shown as the mean ±SEM. *p < 0.05, ***p < 0.001, ****p < 0.0001.

The experimental results indicated that, compared to the CON rats, the protein expressions of VDR, IL-27R, and P38MAPK, as well as the mRNA expressions of IL-27R and PGC-1α, were significantly decreased in the inguinal adipose tissue of HFD rats. In contrast, the protein expressions of PGC-1α and UCP-1, along with the mRNA expressions of VDR and P38MAPK, did not show significant changes (Figures 4A–K). Following VD_3_ intervention, there was a substantial improvement in the protein expressions of VDR, IL-27R, P38MAPK, and UCP-1, and in the mRNA expressions of VDR, IL-27R, P38MAPK, and PGC-1α; additionally, the protein expression of PGC-1α exhibited an upward trend (Figures 4A–K). Therefore, it is suggested that VD_3_ can affect the IL-27/P38MAPK/ PGC-1α pathway, which may enhance lipid deposition in serum and inguinal fat of high-fat diet rats and promote white fat beige.

### 3.5 Calcitriol reduces lipid deposition *in vitro*

**Figure 5:**
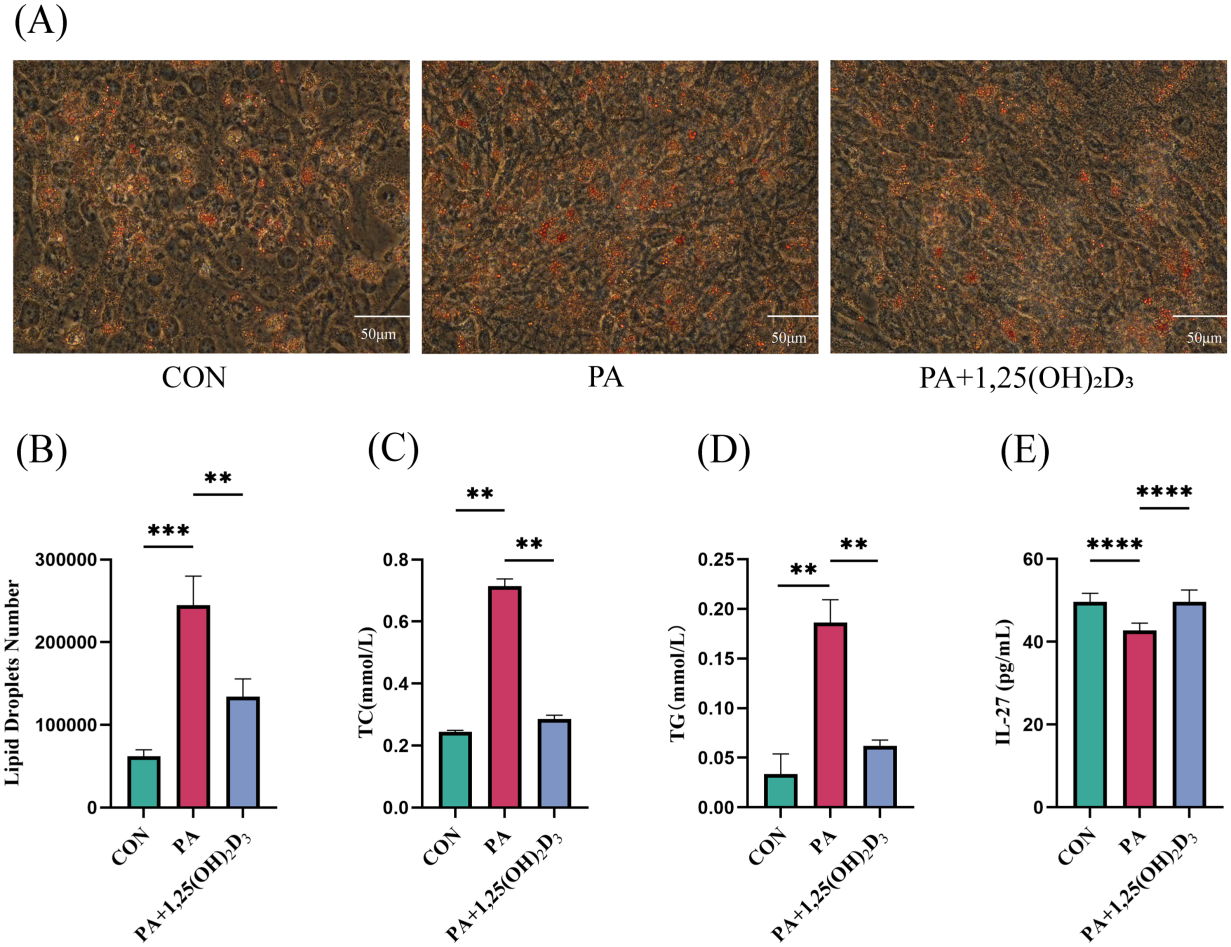
Calcitriol reduces lipid deposition and increases the levels of IL-27 in 3T3-L1 cell. **(A)** Intracellular lipid accumulation was detected by Oil red O staining method using an inverted microscope (400 × magnification). **(B)** Number of LDs. **(C)** Content of TC, **(D)** TG,**(E)** IL-27 in 3T3-L1 cells. Data were shown as the mean ±SEM. *p < 0.05, **p < 0.01, ***p < 0.001.

To investigate the impact of calcitriol on lipid accumulation, an in vitro model of hypertrophic adipocytes was developed. Hypertrophic adipocytes were treated with calcitriol at a concentration of 100 nM. The results indicated that, compared to the control group of mature adipocytes, PA induced a significant accumulation of LD, intracellular TG, and intracellular TC in mature adipocytes. Furthermore, the level of IL-27 was significantly lower in the PA group compared to the CON group. Treatment with 100 nM calcitriol significantly inhibited PA-induced lipid accumulation; the number of LDs in hypertrophic adipocytes was significantly decreased, and the levels of TC and TG were significantly decreased, while the level of IL-27 was significantly increased (Figures 5A–E).

### 3.6 Calcitriol promotes the beige of 3T3-L1 cells

**Figure 6:**
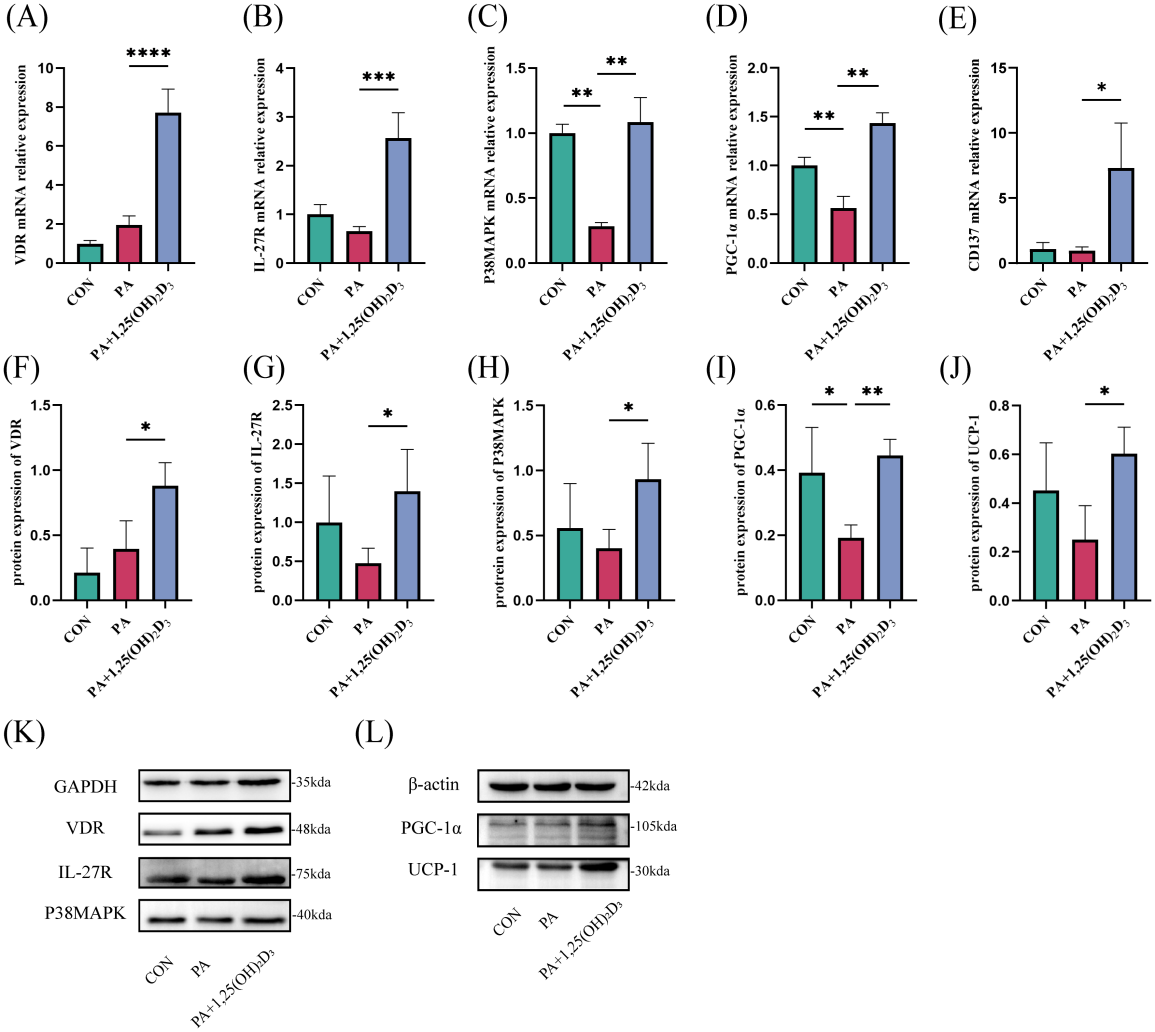
Effects of vitamin D_3_ on the IL-27/P38MAPK/PGC-1α pathway at mRNA and protein levels in 3T3-L1 cells. **(A–E)** The mRNA expression of VDR,IL-27R,P38MAPK,PGC-1α, and CD137 were detected by qPCR. **(F–L)** The protein expression of VDR, IL-27R, P38MAPK, PGC-1α and UCP-1 were detected by western blot, Data were shown as the mean ±SEM. *p < 0.05, **p < 0.01, ***p < 0.001, ****p < 0.0001.

Rt-qPCR and western blot were used to detect the expression levels of IL-27/P38MAPK/PGC-1α pathway-related mRNA and protein. In comparison to the control group, the mRNA and protein expressions of VDR, IL-27R, and the mRNA expression of CD137 in the PA group remained unchanged (Figures 6A,B,E–G). Simultaneously, the mRNA expression of P38MAPK was significantly reduced, while its protein expression did not exhibit significant changes (Figures 6C,H). Additionally, both mRNA and protein expressions of PGC-1α were significantly decreased (Figures 6D,I). A decreasing trend was observed in the protein expression of UCP-1 (Figures 6J,L). Following treatment with 100 nM 1,25(OH)_2_D_3_ for 24 h, the mRNA expression levels of VDR, IL-27R, P38MAPK, PGC-1α, and CD137 were significantly elevated (Figures 6A–E). Simultaneously, the protein expression levels of VDR, IL-27R, P38MAPK, PGC-1α, and UCP-1 also exhibited significant increases (Figures 6F–L).

### 3.7 IL-27 siRNA and PGC-1α siRNA reverse the amelioration of calcitriol in 3T3-L1 cells

**Figure 7:**
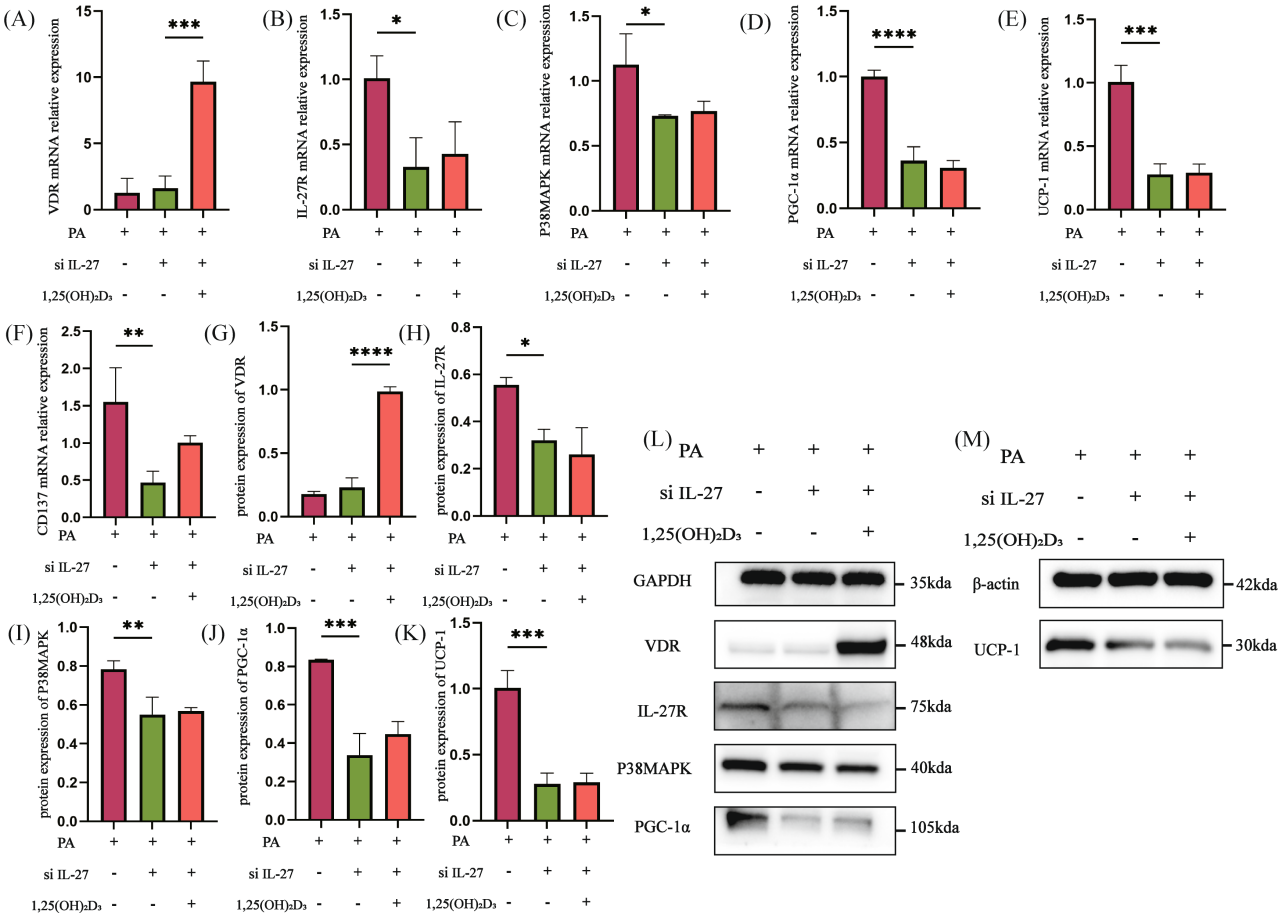
IL-27 siRNA inhibited the effect of Vitamin D_3_. **(A–F)** The mRNA expression of VDR, IL-27R, P38MAPK, PGC-1α, UCP-1 and CD137 were detected by qPCR. **(G–M)** The protein expression of VDR, IL-27R, P38MAPK, PGC-1α, and UCP-1 were detected by western blot, Data were shown as the mean ±SEM. *p < 0.05, **p < 0.01, ***p < 0.001, ****p < 0.0001.

IL-27 siRNA and PGC-1α siRNA were utilized to further explore whether vitamin D_3_ promotes browning of hypertrophic adipocytes by affecting the IL-27/P38MAPK/PGC-1α pathway. Initially, following IL-27 siRNA transfection, the mRNA and protein expressions of VDR in the PA + si IL-27 group were not significantly changed compared to the PA group (Figures 7A,G,L). Conversely, the mRNA and protein expression of IL-27R, P38MAPK, PGC-1α, UCP-1, and the mRNA expression of CD137 were significantly decreased (Figures 7B–F,L,M). After treatment with 100 nM 1,25(OH)_2_D_3_ for 24 h, the mRNA and protein expressions of VDR were markedly increased (Figures 7A,G,L). However, the mRNA and protein expression of IL-27R, P38MAPK, PGC-1α, UCP-1, and the mRNA expression of CD137 remained unchanged (Figures 7B–F,H).

**Figure 8:**
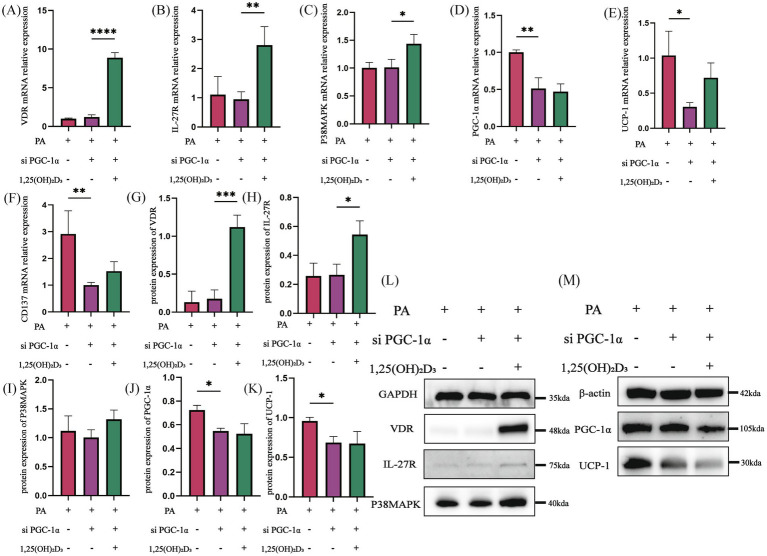
PGC-1α siRNA inhibited the effect of Vitamin D_3_. **(A–F)** The mRNA expression of VDR, IL-27R, P38MAPK, PGC-1α, UCP-1 and CD137 were detected by qPCR. **(G–M)** The protein expression of VDR, IL-27R, P38MAPK, PGC-1α, and UCP-1 were detected by western blot, Data were shown as the mean ±SEM. *p < 0.05, **p < 0.01, ***p < 0.001, ****p < 0.0001.

Then, following PGC-1α siRNA transfection, the mRNA and protein expressions of VDR, IL-27R, and P38MAPK in the PA + si PGC-1α group did not show significant changes compared to the PA group (Figures 8A–C,G). However, the mRNA and protein expressions of PGC-1α, UCP-1, and the mRNA expression of CD137 were significantly decreased (Figures 8D–F,J). After treatment with 100 nM 1,25(OH)_2_D_3_ for 24 h, both the mRNA and protein expressions of VDR and IL-27R were significantly increased (Figures 8A,B,G,H,L). The mRNA expression of P38MAPK was significantly increased, whereas its protein expression did not exhibit significant changes (Figures 8C,I,L). Additionally, the mRNA and protein expressions of PGC-1α, UCP-1 and the mRNA expression of CD137 were not significantly changed (Figures 8D–F,J,K,M). Following IL-27/PGC-1α siRNA transfection, the activity of the IL-27/P38MAPK/PGC-1α pathway was decreased.

## 4 Discussion

Overweight and obesity have become a global epidemic, with prevalence rates demonstrating a sustained upward trajectory worldwide (31). Currently, promoting white fat beige is considered one of the feasible strategies for obesity prevention. Accumulating studies have increasingly stressed the critical role of VD_3_ in the pathogenesis of obesity (32). Previous research has also found that vitamin D_3_ can enhance lipolysis and inhibit lipogenesis (27). This study reveals the significant and essential role of VD_3_ in ameliorating lipid deposition and explores its effects on the IL-27/P38MAPK/PGC-1α pathway.

The level of IL-27 may be associated with VD_3_. Following VD_3_ supplementation, both IL-27 mRNA expression and plasma levels of IL-27 were observed to increase (33). The intervention with VD_3_ and/or RSV alleviated metabolic abnormalities induced by type 2 diabetes mellitus (T2DM), resulting in elevated levels of the anti-inflammatory factor IL-27 and reduced levels of the pro-inflammatory factor IL-23 in the hippocampus of rats (34). Additionally, the expression of IL-27 in the spinal cord of experimental autoimmune encephalomyelitis (EAE) mice may be enhanced by vitamin D (35). Consistent with these findings, our study revealed that after VD_3_ intervention, the levels of IL-27, along with the mRNA and protein expressions of IL-27R, significantly increased in HFD rats and 3T3-L1 cells. Notably, when IL-27 was knocked down in 3T3-L1 cells, the mRNA and protein expressions of IL-27R did not show significant changes following VD_3_ intervention.

Prior research has demonstrated that multiple signaling pathways are involved in white fat beige. Following the activation of β3-adrenergic receptors, the cAMP-PKA (protein kinase A) signaling pathway is triggered, leading to the activation of downstream P38 kinase and ERK1/2. This process promotes the transformation of white adipocytes into beige adipocytes and enhances their thermogenic capacity (36). Additionally, some bioactive substances promote white fat beige by activating the PI3K-ERα signaling pathway (37). Naringenin has been shown to reduce lipid accumulation via the AMPK pathway (38). Furthermore, astragalin and rutin modulate PPAR-*γ* and AMPK-mediated signaling pathways to alleviate obesity disorders (39). In studies involving 3T3-L1 adipocytes and mice fed a high-fat diet deficient in vitamin D, vitamin D_3_ was found to inhibit the activation of the PI3K/Akt/mTOR signaling pathway, thereby suppressing the expression of browning markers in white adipocytes, such as PPAR-γ, PGC-1α, and UCP-1 (40). The synergistic effect of these signaling pathways plays a crucial role in regulating energy metabolism and lipid storage in adipocytes, potentially contributing to obesity prevention.

A positive correlation between 25(OH)_2_D_3_ and IL-27 was observed in both the observation and control groups. The IL-27/P38MAPK/PGC-1α pathway is closely associated with inflammation and white fat beige. Meanwhile, calcitriol can inhibit the p38 MAPK signaling pathway by up-regulating MAPK phosphatase-5 (MKP-5), thereby reducing the production of pro-inflammatory cytokines such as IL-6 and TNF (41). One study demonstrated that the IL-27/P38MAPK/PGC-1α pathway could promote thermogenesis and energy expenditure of adipocytes (9). Therefore, this study hypothesizes that the molecular mechanism by which VD_3_ promotes white fat beige may be through this pathway. Subsequently, the mRNA and protein expression levels of the IL-27/P38MAPK/PGC-1α pathway, as well as its upstream and downstream genes, were assessed using 3T3-L1 cells and rat inguinal adipose tissue.

**Figure 9:**
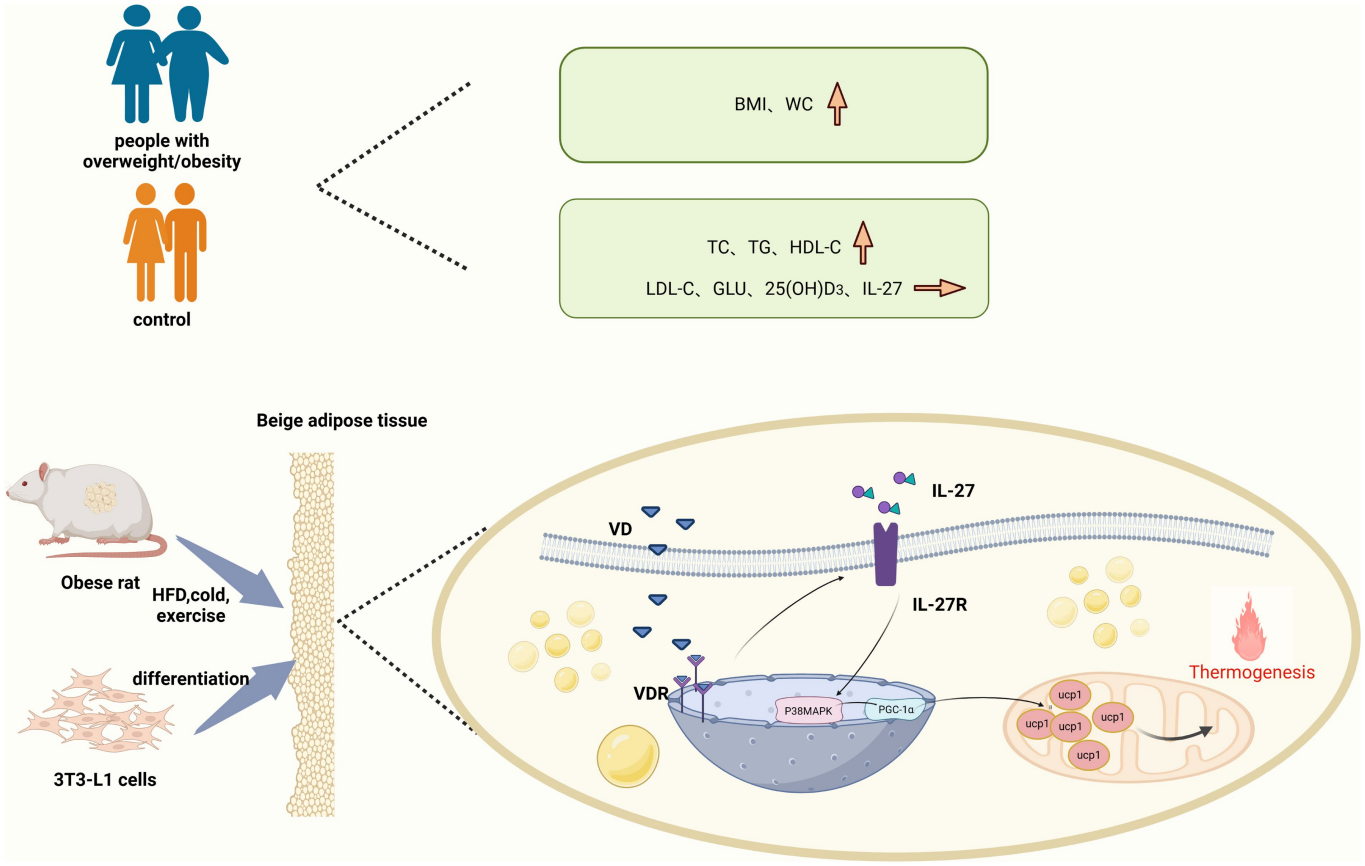
White fat beige machine drawing.

*In vivo* experiments demonstrated that VD_3_ intervention significantly upregulated the expression of VDR, IL-27R, and P38MAPK at both mRNA and protein levels compared to the HFD group. Additionally, PGC-1α mRNA expression showed an increase; however, no significant difference in protein expression was observed between the groups. The results may be attributed to several factors: First, mRNA stability is inherently low, leading to rapid degradation that hinders effective protein synthesis. Second, the process of mRNA-to-protein translation is inherently limited. Finally, the transcription and translation of mRNA are tightly coupled processes; if this coupling is disrupted, mRNA levels may increase while protein levels remain unchanged. Notably, UCP-1 protein expression in inguinal adipose tissue also significantly increased following VD_3_ intervention, with similar results observed in mast cells. The intervention of 1,25(OH)_2_D_3_ in 3T3-L1 cells enhanced both mRNA and protein expression of VDR, IL-27R, P38MAPK, and PGC-1α compared to the PA group. Surprisingly, UCP-1 protein levels and CD137 mRNA levels in mast cells were significantly elevated after VD_3_ intervention. However, both IL-27 siRNA and PGC-1α siRNA inhibited the beneficial effect of VD_3_ in 3T3-L1 cells. Therefore, the relationship between VD_3_ and IL-27 may involve VD_3_ binding to VDR upon entering the body, which stimulates the secretion of IL-27. Consequently, P38MAPK/PGC-1α, as downstream targets of IL-27, are mobilized to further enhance the levels of PGC-1α, UCP-1, and CD137, which serve as markers for beige fat (Figure 9). This suggests that VD_3_ intervention may stimulate the generation of the thermogenic gene UCP-1 and CD137 via the IL-27/P38MAPK/PGC-1α pathway, thereby increasing adaptive thermogenesis, promoting white fat beige, and reducing lipid accumulation.

However, in this small sample population study, no significant differences were observed in the serum 25(OH)D_3_ levels and serum IL-27 levels between the two groups. The results may be attributed to the following points: First, vitamin D is primarily synthesized in the skin upon exposure to ultraviolet rays (42). An intervention trial indicated that serum 25(OH)D_3_ levels increased from 36.4 nmol/L in winter to 49.8 nmol/L in summer, subsequently decreasing to 39.6 nmol/L in winter when relying solely on natural sunlight exposure (43). The recruitment, sampling, and testing of participants in this study were conducted exclusively in summer, a season characterized by relatively abundant ultraviolet rays, during which the levels of vitamin D in the human body are typically elevated and stable (44). Secondly, an individual’s daily activity level and clothing choices can significantly impact sunlight exposure, thereby influencing the levels of vitamin D in the body (45). This study did not collect or analyze information regarding the participants’ outdoor exercise duration, frequency, or sun protection habits. Previous population-based case–control studies have reported that the observation group exhibited increased serum IL-27 levels compared to the normal control group, which contradicts the findings of this study. Several factors may contribute to these discrepancies. Firstly, the participants in this study were primarily college students and a few teachers, representing a specific subgroup characterized by a higher prevalence of simple obesity and fewer comorbidities. Secondly, the participants in this study had a BMI of 27.09 kg/m^2^, which is significantly lower than the BMI of 38.92 kg/m^2^ reported in another study (9). Higher BMI is often associated with greater adiposity and metabolic dysfunction, making it more likely to observe significant differences between groups with a larger BMI disparity (46, 47). The lack of difference in 25(OH)D_3_ between the two groups may also be attributed to smaller differences in BMI (48). This study conducted a correlation analysis between 25(OH)D_3_ and IL-27 levels in all participants, which revealed a positive correlation.

The level of serum 25(OH)D_3_ was reduced after VD_3_ intervention in HFD rats. This reduction may be attributed to corn oil, the lipid carrier for vitamin D supplements. In the study conducted by Du et al., it was observed that the serum 25(OH)D_3_ level in the HFD + oil group was significantly lower than that in the HFD group. Conversely, the serum 25(OH)D_3_ level in the HFD + VD group was significantly higher than that in the HFD + oil group when vitamin D was dissolved in corn oil and administered via gavage (49). Furthermore, a randomized controlled trial demonstrated that the choice of lipid carriers for vitamin D supplementation influences serum 25(OH)D_3_ levels (50). Vitamin D_3_, derived from dietary sources and from 7-dehydrocholesterol (7DHC) present in the skin, is first hydroxylated to 25(OH)D_3_ in the liver. This compound is then converted to 1,25(OH)_2_D_3_ via the enzyme CYP27B1 in the kidneys. Corn oil, which is abundant in vitamin E, serves as an antioxidant. Additionally, it has been shown that rosemarinic acid (RA), another antioxidant, specifically enhances the enzymatic activity of peroxidase 1 (PRDX1), thus alleviating nonalcoholic steatohepatitis (NASH) (51). The higher levels of unsaturated fatty acids in corn oil may interact with vitamin D metabolism, potentially influencing metabolic processes in the body (52). These factors may indirectly hinder the conversion of vitamin D_3_ in the liver, ultimately leading to lower serum 25(OH)D_3_ levels.

In a previous study, it was demonstrated that VD_3_ intervention resulted in significantly higher levels of inflammatory cytokines, such as IL-6, MCP-1, and TNF-*α*, compared to the control group (27). In *in vivo* experiments, VD_3_ intervention significantly reversed the HFD-induced upregulation of serum inflammatory cytokines IL-6 and MCP-1. However, there was no significant improvement in TNF-α levels, which is inconsistent with the findings of Cordeiro et al. (53). This discrepancy may be attributed to the use of different rat models; Cordeiro et al. employed an experimental model that more closely resembles the obesogenic conditions found in humans (53). Obesity is associated with the accumulation of inflammatory immune cells in white adipose tissue, and white adipose beige was suppressed when the body was in an inflammatory state (54, 55). The findings suggest that VD_3_ may promote white fat beige by decreasing the expression of inflammatory factors within adipocytes.

Intervention of VD_3_ in these rats reduced energy intake, without substantial fluctuations in body weight. The study conducted by Zhang et al. (56) supports the notion that substantial changes in body weight may not occur solely with VD_3_ intervention. Supports the notion that significant changes in body weight may not occur with only VD_3_ intervention. While VD_3_ alone may have no significant impact on weight, it may still play a vital role in modulating various metabolic pathways that contribute to obesity development (57). By targeting these pathways and influencing obesity-related biomarkers, VD_3_ intervention could potentially contribute to obesity reduction (58, 59). These findings are consistent with previously reported studies suggesting that VD_3_ intervention effectively mitigates obesity (60, 61).

The level of HDL-C was also reduced after VD_3_ intervention in HFD rats. Previous studies have reported similar findings, indicating that serum HDL-C levels decrease after VD_3_ intervention in mice (26). A meta-analysis also revealed that VD supplementation did not significantly improve HDL-C levels in patients with NAFLD (62). The formation of new HDL-C involves the secretion of apolipoprotein A-I by the liver and small intestine (63). HDL-C particles not only transport cholesterol from various cells but also incorporate proteins, hormones, and vitamins (64). In the context of a diet high in fats and cholesterol, mice may develop NAFLD (49). During this condition, lipids accumulate within the liver, leading to impaired lipid metabolism regulation (65). Following VD_3_ intervention, there is an accelerated process of HDL-C transport, while the secretion of HDL-C by the liver is diminished (66). This reduction in liver HDL-C secretion may contribute to the abnormal decrease in HDL-C levels observed after VD_3_ intervention.

This study has several limitations. Firstly, the population only has cross-sectional data. Vitamin D_3_ exhibits significant potential in obesity prevention; nonetheless, its efficacy warrants additional assessment in forthcoming clinical research. Secondly, the *in vivo* experiments were restricted to male rats; future research should include female subjects. Thirdly, IL-27 siRNA and PGC-1α siRNA were used in this study, whereas gene overexpression and silencing methods *in vitro* and in vivo are needed in future research. Finally, VD_3_ may also reduce lipid accumulation and promote energy expenditure through pathways other than the IL-27 pathway. Therefore, whether VD_3_ is a mediator or a cause of obesity treatment needs to be further investigated.

## 5 Conclusion

In conclusion, VD_3_ promotes white fat beige by elevating IL-27 levels, an effect potentially linked to the IL-27/P38MAPK/PGC-1α pathway.

## Data Availability

The original contributions presented in the study are included in the article/Supplementary material, further inquiries can be directed to the corresponding authors.
