# Supplementary material for: Vitamin D3 promotes white fat beige through IL-27/P38MAPK/PGC-1α pathway
Source: Front Nutr. 2025 Sep 18;12:1661072. doi: 10.3389/fnut.2025.1661072 (PMC12488405; doi:10.3389/fnut.2025.1661072)
Supplement: Supplementary file 1 [file Table_1.docx]

| Gene | Forward primer  (5′–3′) | Reverse primer  (5′–3′) |
| --- | --- | --- |
| β-actin | CACCATGTACCCAGGCATTG | CCTGCTTGCTGATCCACATC |
| VDR | AGAGGTGTTCGGCAATGAGA | TGGGCAGGAGAAAGGATGAG |
| IL-27R | CACCAGAGTCAGAAATACCATCCCA | CCAGATGAGGAGTTTGTCAGCCATG |
| P38MAPK | TTACCGATGACCACGTTCAGTTTC | AGCGAGGTTGCTGGGCTTTA |
| PGC-1α | TATGGAGTGACATAGAGTGTGC | CCACTTCAATCCACCCAGAAAG |
| UCP-1 | TTGGGCTTCTATGCTGGGAG | GTGAATGCTATGCTCTTCTGTCT |
| CD137 | GGTCTGTGCTTAAGACCGGG | TCTTAATAGCTGGTCCTCCCTC |

Table S1 The sequences of the qPCR primer
